# Diagnostic Accuracy of Computed Tomography for Chronic Thromboembolic Pulmonary Hypertension: A Systematic Review and Meta-Analysis

**DOI:** 10.1371/journal.pone.0126985

**Published:** 2015-04-29

**Authors:** Chengjun Dong, Min Zhou, Dingxi Liu, Xi Long, Ting Guo, Xiangquan Kong

**Affiliations:** Department of Radiology, Union Hospital, Tongji Medical College, Huazhong University of Science and Technology, Wuhan, Hubei, People’s Republic of China; University of Dundee, UNITED KINGDOM

## Abstract

This study aimed to determine the diagnostic accuracy of computed tomography imaging for the diagnosis of chronic thromboembolic pulmonary hypertension (CTEPH). Additionally, the effect of test and study characteristics was explored. Studies published between 1990 and 2015 identified by PubMed, OVID search and citation tracking were examined. Of the 613 citations, 11 articles (n=712) met the inclusion criteria. The patient-based analysis demonstrated a pooled sensitivity of 76% (95% confidence interval [CI]: 69% to 82%), and a pooled specificity of 96% (95%CI: 93% to 98%). This resulted in a pooled diagnostic odds ratio (DOR) of 191 (95%CI: 75 to 486). The vessel-based analyses were divided into 3 levels: total arteries、main+ lobar arteries and segmental arteries. The pooled sensitivity were 88% (95%CI: 87% to 90%)、95% (95%CI: 92% to 97%) and 88% (95%CI: 87% to 90%), respectively, with a pooled specificity of 90% (95%CI: 88% to 91%)、96% (95%CI: 94% to 97%) and 89% (95% CI: 87% to 91%). This resulted in a pooled diagnostic odds ratio of 76 (95%CI: 23 to 254),751 (95%CI: 57 to 9905) and 189 (95%CI: 21 to 1072), respectively. In conclusion, CT is a favorable method to rule in CTEPH and to rule out pulmonary endarterectomy (PEA) patients for proximal branches. Furthermore, dual-energy and 320-slices CT can increase the sensitivity for subsegmental arterials, which are promising imaging techniques for balloon pulmonary angioplasty (BPA) approach. In the near future, CT could position itself as the key for screening consideration and for surgical and interventional operability.

## Introduction

Chronic thromboembolic pulmonary hypertension (CTEPH) is a life threatening complication and carries poor diagnosis[1]. It is characterized by obstruction of the large pulmonary arteries by acute and recurrent pulmonary emboli[2]. These changes lead to a progressive elevation of pulmonary arterial pressure (PAP) and pulmonary vascular resistance (PVR) and then subsequent right-sided heart failure[3]. Recent studies suggest that the incidence of CTEPH varies from 1% to 3.8% of patients 2 years after acute pulmonary embolism (APE)[4, 5], and CTEPH has a poor prognosis without surgical intervention[6]. Pulmonary endarterectomy (PEA) and BPA has been demonstrated feasible for the treatment of CTEPH. However, the choice of therapy predominantly depends on the presence of inaccessible distal disease [7]. Therefore, preoperative workup is crucial to determine if it is eligible to perform either PEA or BPA[8]. Ventilation/perfusion (V/Q) lung scintigraphy used to be recommended to differentiate CTEPH from unexplained pulmonary hypertension (PH)[9]. There was consensus among experts that the presence of multiple bilateral perfusion defects with normal ventilation allowed the diagnosis of CTEPH. Nevertheless, some trials have depicted that other types of PH including pulmonary veno-occlusive disease, fibrosing mediastinitis, and sarcomas of the pulmonary arteries may occasionally appear the same abnormalities[10, 11]. Combining with right heart catheterization, selective digital subtraction angiography (DSA) remains the choice of diagnosing CTEPH and assessing the eligibility of surgery[12]. But it's invasive and expensive which makes DSA reluctant to be widely used[13]. With the advent of multidetector-row computed tomography (MDCT), computed tomography (CT) has also being increasingly used in the diagnosis of CTEPH[14]. However, uncertainty still persists about the performance of CT for the diagnosis of CTEPH. Therefore it is necessary to know the diagnostic performance of CT for CTEPH. The aim of the study is to conduct a meta-analysis to clarify the role of CT for CTEPH and furthermore provides guidance to clinicians regarding imaging management of CTEPH.

## Materials and Methods

### Data Sources and Study Selection

This meta-analysis was conducted in accordance with the Preferred Reporting Items for Systematic reviews and Meta-Analyses (PRISMA) guideline (S1 Table). We searched the PubMed database and Ovid database for English literature from January 1990 to January 2015 on the diagnostic accuracy of CT for the detection of CTEPH. Both free-text searching and thesaurus terms (Medical Subject Headings terms for Pubmed and EMTREE for OVID) were used. We combined search terms for applied technique (Computed Tomography) and disease (Chronic Thromboembolic Pulmonary Hypertension). The bibliographies of included studies and reference lists were also screened for potentially suitable studies. We included a study if: 1) CT was used as a diagnostic test for CTEPH; 2) Patients were referred for suspected or confirmed CTEPH; 3)DSA and/or V/Q scanning were used as reference standard; 4) absolute numbers of true positive, false positive, true negative, and false negative cases were given, or if these data were derivable from the presented results. A study was eligible regardless of what CT technique was used. Studies were excluded if they were: 1) reviews or case reports; 2) conducted with animals. Two investigators independently performed the screening, and any discrepancy was resolved through discussion or by involving a third reviewer (XK), when necessary.

QUADAS-2 tool was used to assess the quality of studies included. We assumed that only V/Q scanning or DSA could correctly rule in or rule out CTEPH.

### Data Extraction

First, identifying information about the study such as first author, journal, and year of publication was extracted (Table 1). Further extracted variables consisted of patient characteristics, technical information and absolute numbers of true negative, true positive, false negative, and false positive test results (Table 2). If available, data were recorded on patient basis and vessel basis (i.e., main + lobar pulmonary arteries and segmental arteries). Two investigators (C.D and M.Z) extracted data independently, and discrepancies were resolved by consensus.

**Table 1:** Characteristics of included studies^1^.

Author	Year	Study Size (Male, %)	Mean Age, y	Prevalence (%)	Time Interval	Selection	Slices	Contrast Material	Reference Standard	mPAP	SPAP
Bartalena	2008	39(36.4%)	59	34.6	0-86d	Confirmed PH	16	Iomeron	V/Q,CTPA	Unclear	Unclear
He	2012	49(43%)	43.3	44.7	7d	Suspected CTEPH	16 or 64	Unclear	DSA	52.77±18.31	1474.66±671.16
Dournes	2014	14(35%)	67.1	35	2.0±3.8d	Confirmed PH	DECT	Xenetix 350	V/Q	Unclear	Unclear
Tunariu	2007	85(37.4%)	42	34.8	48h-10d	Confirmed PH	Unclear	Unclear	DSA,V/Q,CTPA	Unclear	Unclear
Sugiura	2013	16(36%)	59.2	100	2d-2w	Suspected CTEPH	320	Iomeron	DSA	42.2±9.9	696±274
Reichelt	2009	13(48%)	59	89	2d-2w	Suspected CTEPH	64	Imeron	DSA,V/Q	46±8	763±345
Ley	2012	13(54.2%)	58	100	3d	Suspected CTEPH	40 or 64	Imeron	DSA	42±10	Unclear
Soler	2011	5(55.6%)	52.8	100	Unclear	Confirmed CTEPH	4 to 64	Unclear	DSA	40±8	450±178
Nakazawa	2011	35(67%)	57.7	100	2.5d	Suspected CTEPH	DECT	Unclear	DSA	Unclear	Unclear
Bergin	1997	Unclear	Unclear	87	Unclear	Suspected CTEPH	Unclear	Ioversol	DSA	Unclear	Unclear
Zhang	2013	Unclear	49	100	10d	Confirmed CTEPH	64	Unclear	DSA	Unclear	Unclear

^1^mPAP: mean PAP; sPAP: standard PAP.

**Table 2:** Absolute numbers of included studies^1^.

Author	Patient-Based Analysis					Vessel-Based Analysis														
						Total Vessels					Main + Lobar					Segmental				
	No.	TP	FN	FP	TN	No.	TP	FN	FP	TN	No.	TP	FN	FP	TN	No.	TP	FN	FP	TN
Bartalena	107	35	2	7	63															
He	114	47	4	3	60															
Dournes	40	14	0	2	23															
Tunariu	227	40	38	1	148															
Sugiura						1175	228	29	44	903	344	65	2	8	269	860	163	27	36	634
Reichelt						724	365	18	23	336	212	109	2	5	96	530	256	16	18	240
Ley						639	370	0	1	268	190	91	0	0	99	449	279	0	1	169
Bergin						910	526	124	102	282	188	43	13	11	121	846	483	111	91	161
Soler						107	67	73	8	32										
Nakazawa						817	686	25	33	98										
Zhang						589	217	51	42	279										

^1^ No.: number; TP: true positive; FN: false negative; FP: false positive; TN: true negative.

### Statistical Analysis

Based on the results from the derived contingency tables, pooled sensitivity, specificity and DOR were calculated. The generated output of sensitivity and 1-specificity were used for the construction of summary receiver operating characteristic (SROC) curves. Also, the area under the curve (AUC) with 95%CIs was computed. SROC curves would generate a composite statistic which can demonstrate the diagnostic ability of CT[15]. The I^2^ index was used to test for the heterogeneity between study results, which described the significance of total variation across studies rather than chance[16]. Statistical heterogeneity was defined as an I^2^ statistic value of >50%. Differences in study characteristics between articles can be a cause of considerable heterogeneity (e.g., the use of different contrast media, PAP and PVR). Therefore, the study characteristics were compared using the meta-regression to test for the sources of heterogeneity. Additionally, pooled estimates were calculated for subgroups of studies that were defined according to specific study characteristics on vessel-based analysis. Furthermore, sensitivity analysis were also performed to assess the reliability and stability of the meta-analysis results. Meta-Disc 1.4 was used for data analysis. Publication bias was examined using Deeks' test[17].

### Clinical practice perspectives

Bayes's theorem was used to calculate the post-test probability of CT on patient-based analysis[18]. We presumed that a post-test probability of 85% and 5% was accurate enough to diagnose and exclude CTEPH, respectively[19]. As for vessel-based analysis, positive predictive value (PPV) and negative predictive value (NPV) were computed.

## Results

### Study Identification

**Fig 1:**
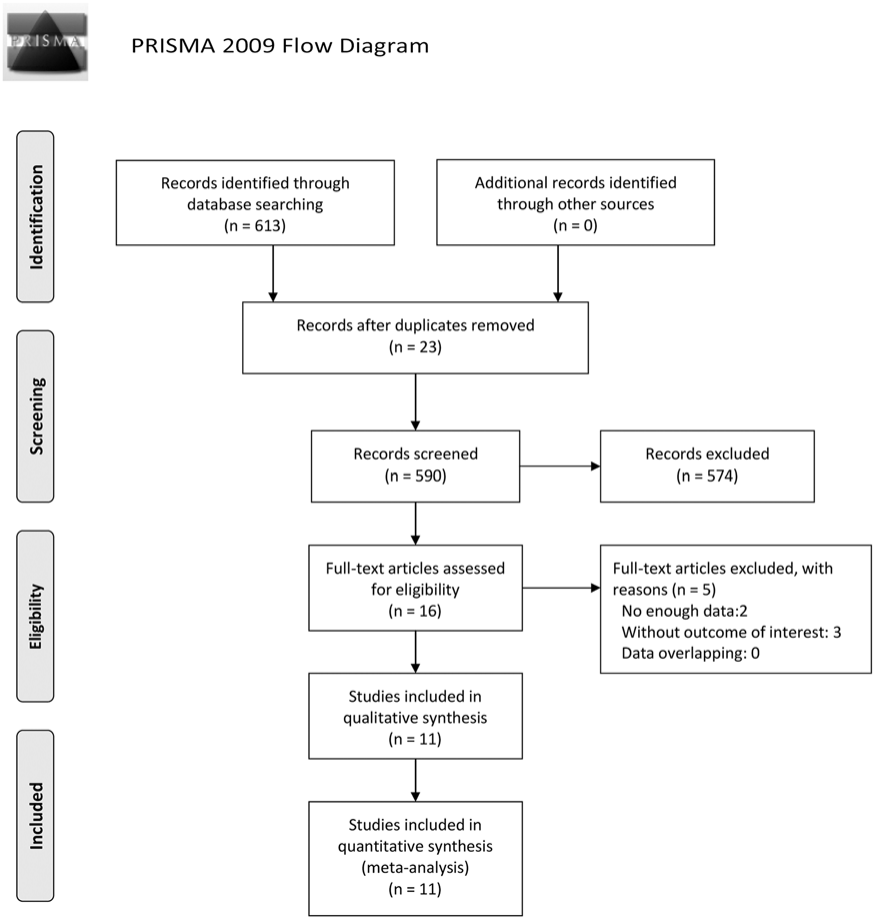
Flow Diagram of Literature Search. Of 613 potentially relevant citations, 11 articles met our inclusion criteria and were included in the meta-analysis. *From*: Moher D, Liberati A, Tetzlaff J, Altman DG, The PRISMA Group (2009). *P*referred *R*eporting *I*tems for *S*ystematic Reviews and *M*eta-*A*nalyses: The PRISMA Statement. PLoS Med 6(6): e1000097. doi:10.1371/journal.pmed1000097 For more information, visit www.prisma-statement.org.

The search process and results were shown in the flow chart in Fig 1. A total of 11 articles met our inclusion criteria: 4 on patient basis and 7 on vessel basis.

### Methodological Quality of Included Studies

**Fig 2:**
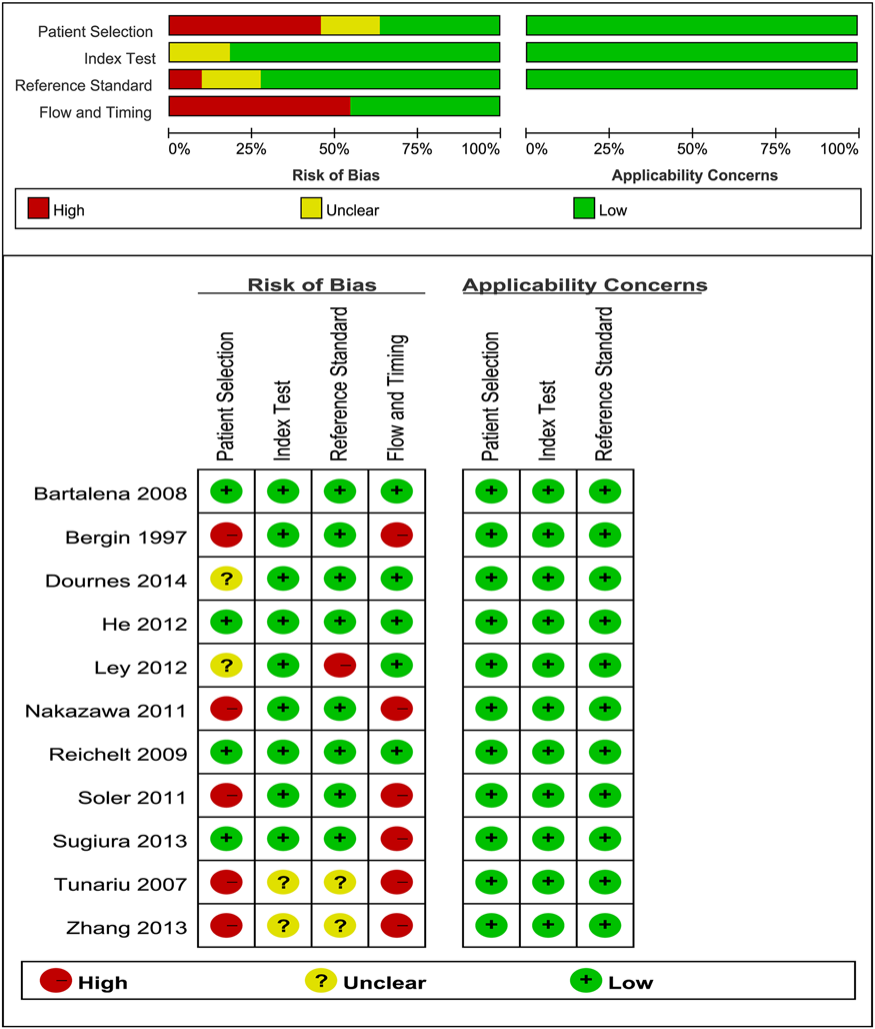
Pooled Quality Assessment of Primary Studies.

Methodological quality of the studies according to the QUADAS-2 tool was summarized in Fig 2.

### Study characteristic

Baseline characteristics of patients included in the studies were summarized in Table 1. In total, 11 articles were retrieved [1, 13, 20–28]. Study size ranged from 9 to 227 patients, with a total of 712 subjects, of whom patient-based and vessel-based analyses included 488 and 224 patients, respectively. We observed a mean prevalence of 37% for CTEPH in patient-based studies. The diagnostic performance of two investigators was reported in one study[27]. Since the agreement between the two readers was excellent, we chose values provided by the first reader.

### Diagnostic Performance of CT

**Fig 3:**
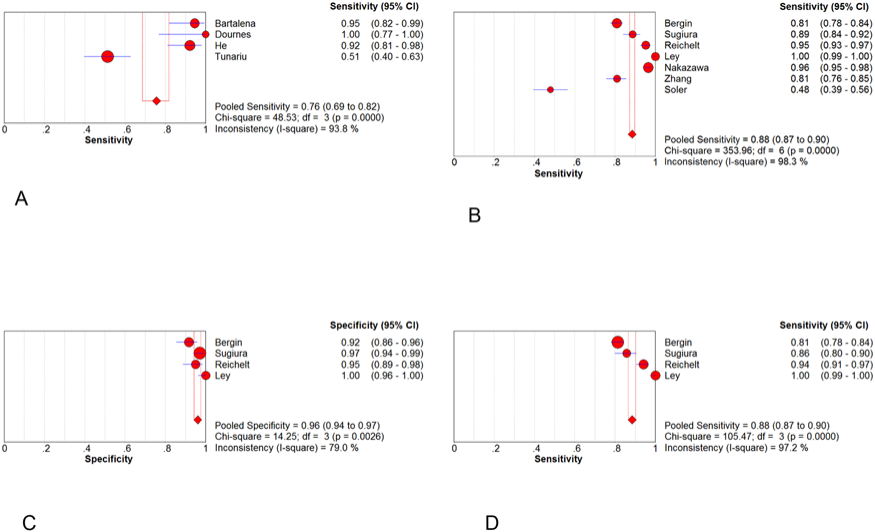
Forest plots of sensitivity. Sensitivity in (A) patient basis; (B) total arteries; (C) main + lobar arteries; (D) segmental arteries.

**Fig 4:**
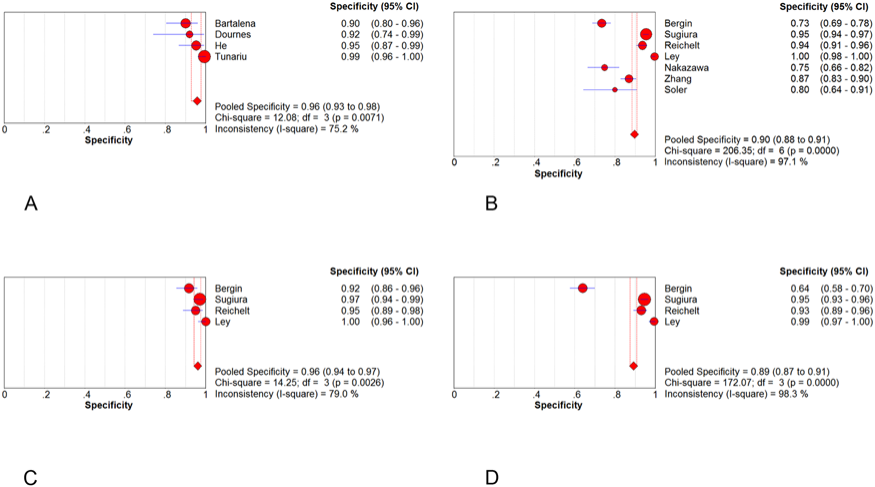
Forest plots of specificity. Specificity in (A) patient basis; (B) total arteries; (C) main + lobar arteries; (D) segmental arteries.

**Fig 5:**
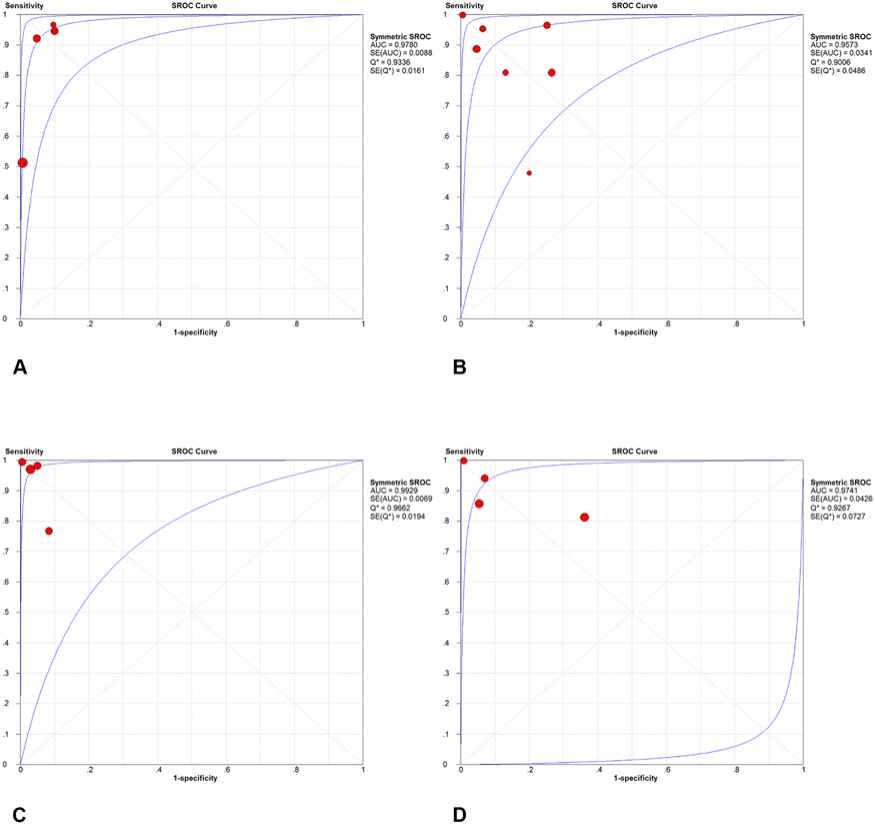
Summary ROC curve for CT. Diagnostic performance of CT in (A) patient basis; (B) total arteries; (C) main + lobar arteries; (D) segmental arteries.

**Fig 6:**
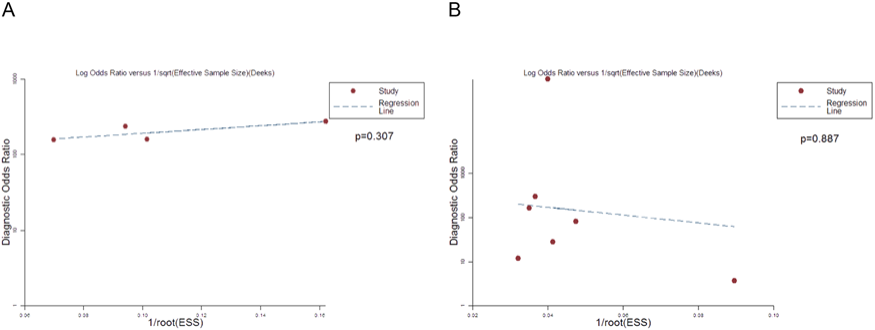
Probabilities of CTEPH after CT on a patient-based level. The plot showed that CT has a favorable positive post-test probability over a wide range of pre-test probabilities, which can be applied to rule in CTEPH.

Forest plots of sensitivity and specificity were shown in Figs 3 and 4. The patient-based analyses demonstrated a moderate sensitivity of 76% (95% CI: 69% to 82%, I^2^ = 93.8%, p<0.01) and a high specificity of 96% (95%CI: 93% to 98%, I^2^ = 75.2%, p<0.01). The vessel-based analyses were divided into three levels: total arteries、main+ lobar arteries and segmental arteries. The more peripheral areas the emboli located, the lower the sensitivity and specificity were. The pooled sensitivity were 88% (95%CI: 87% to 90%)、95% (95%CI: 92% to 97%) and 88% (95%CI: 87% to 90%), respectively. And the pooled specificity were 90% (95%CI: 88% to 91%)、96% (95%CI: 94% to 97%) and 89% (95% CI: 87% to 91%), correspondently. SOC curves of CT on both patient and vessel basis were in Fig 5. The area under the curves was 0.9780, 0.9573, 0.9929 and 0.9741, respectively. There was significant heterogeneity across the studies on both patient and vessel basis. Subgroup analyses were performed to explore all possible sources of heterogeneity (Table 3). The analyses revealed no significant effect of study characteristics on the diagnostic performance of CT, except for a lower pooled DOR of CT in poor-quality studies compared with high-quality studies in overall arterials on vessel basis. We classified studies into 2 categories depending upon the methodological quality: high-quality studies (Ref. 19, 23, 24) and poor-quality studies (Ref. 1, 22, 26, 27). A study was judged poor-quality if it was judged "high risk of bias" in 2 or more domains. Additionally, in vessel level, promising CT (320-slices CT and dual-energy CT) demonstrated a higher but non-significant DOR in comparison to routine CT (64 or less slices CT). Sensitivity analysis demonstrated that pooled estimate did not change with exclusion of any 1 study (Table 4). The post-test probability on patient-based analysis showed that CT has a favorable positive post-test probability over a wide range of pre-test probabilities, which can be applied to rule in the disease (Fig 6). The value of PPV and NPV was 90.6% and 87% in total arteries, 92.8% and 97% in main + lobar arteries and 89% and 89% in segmental arteries, respectively.

**Table 3:** Subgroup analyses for the diagnostic performance of CT on overall arterials level^c^.

Characteristic	Studies, n	Sensitivity % (95% CI)	Specificity % (95% CI)	DOR (95% CI)	RDOR^1^	p Value
Patient selection						
confirmed CTEPH	2	70 (65–74)	86 (82–90)	10 (1–78)	1.00	
suspected CTEPH	5	96 (95–97)	92 (90–94)	349 (73–1671)	35.64	0.18
CT technology						
Promising CT	2	97(95–98)	90(87–93)	268(20–3545)	1.00	
Routine CT	4	79(76–83)	90(87–93)	126(13–1261)	0.46	0.85
Quality						
Poor-quality	4	86(84–88)	85(82–88)	24(8–75)	1.00	
High-quality	3	99(96–100)	97(95–99)	1604(392–6568)	0.46	0.0139

^1^RDOR: relative diagnostic odds ratio.

**Table 4:** Sensitivity analysis on both patient and vessel basis.

Author	Studies, n	Sensitivity%(95%CI)	Specificity%(95% CI)	DOR(95%CI)	p Value
Patient-based	4	76(69–82)	96 (93–98)	191 (75–486)	
Excluding Bartalena	3	71 (62–78)	98 (95–99)	210 (67–657)	0.7408
Excluding He	3	79 (60–77)	96 (93–98)	170 (53–548)	0.809
Excluding Dournes	3	74 (66–80)	96 (93–98)	184 (69–490)	0.8774
Excluding Tunariu	3	94 (88–98)	92 (87–96)	202 (70–579)	0.847
Vessel-based	7	88 (87–90)	90 (88–91)	76 (23–254)	
Excluding Sugiura	6	88 (86–90)	88 (86–90)	70 (20–247)	0.4649
Excluding Reichelt	6	88 (86–89)	90 (88–92)	72 (20–258)	0.6972
Excluding Ley	6	88 (85–89)	89 (87–91)	71 (21–241)	0.1029
Excluding Bergin	6	89 (87–91)	90 (89–92)	144 (32–656)	0.8667
Excluding Soler	6	93 (91–94)	91 (89–92)	185 (56–614)	0.2795
Excluding Nakazawa	6	81 (78–84)	92 (91–94)	132 (25–687)	0.0909
Excluding Zhang	6	90 (88–92)	92 (90–94)	171 (29–996)	0.6446

### Publication Bias

**Fig 7:**
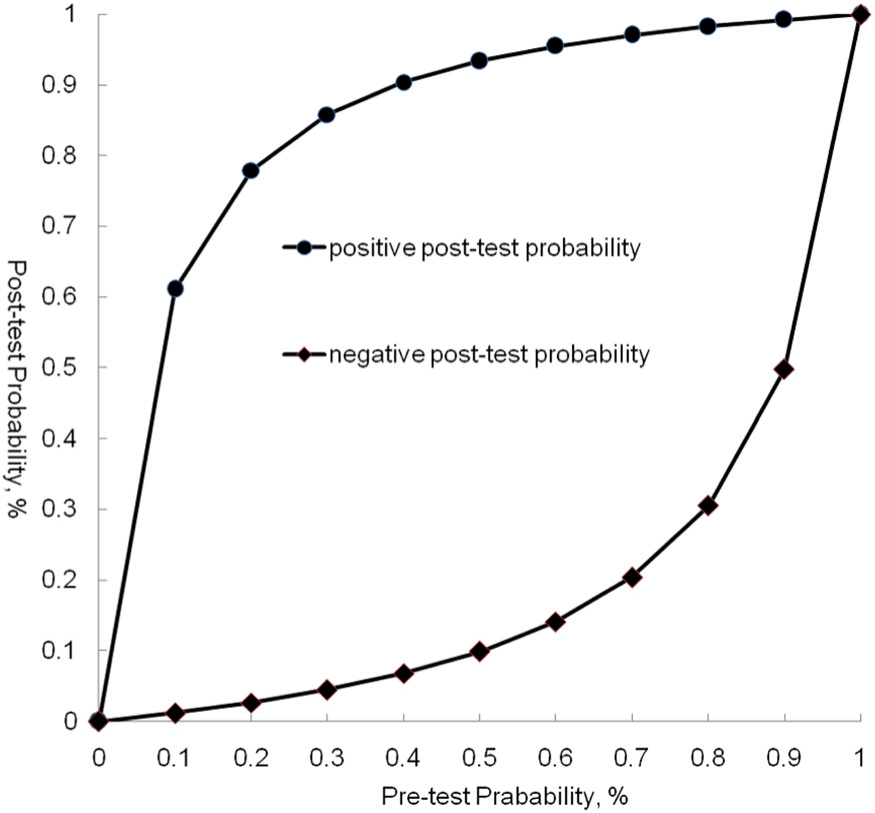
Deek’s test to measure the funnel plot asymmetric. P-values less than 0.05 were considered representative of publication bias. (A) patient basis; (B) total arteries.

The effective sample size funnel plot and associated regression test of asymmetry (Deeks 2005) were used to detect publication bias[17]. The results were shown in Fig 7. There was no evidence of publication bias in both patient-based and vessel-based analysis.

## Discussion

There are three challenges in imaging of patients with suspected CTEPH: an accurate diagnostic performance of the imaging modality is essential for the presence of CTEPH. It should enable detection of a patient with confirmed CTEPH suitable for PEA with great certainty, and finally, allow the adequate quantification of PH by measuring pulmonary hemodynamics[29]. Several imaging modalities can be applied as diagnostic tools for CTEPH, inclusive of DSA, V/Q lung scintigraphy, multidetector CT angiography and magnetic resonance angiography[2, 30]. Nowadays, invasive pulmonary DSA is a standard diagnostic tool used to assess patients with suspected CTEPH for both establishing the diagnosis and evaluating operability [31, 32]. In contrast to pulmonary DSA, CT is a non-invasive substitute for pulmonary vascular imaging[21]. And it has been found beneficial to determine the causes of PH, affect the selection of surgical methods and predict prognosis after surgery[33]. Accumulating number of articles has focused on the diagnostic accuracy of CT for CTEPH. To the best of our knowledge, this is the first meta-analysis carried out to estimate the role of CT in detection of CTEPH.

Our results indicated a moderate sensitivity and a high specificity of CT for the assessment of CTEPH on patient basis. However, in vessel-based analysis, our meta-analysis revealed both a satisfactory sensitivity and specificity, especially at the main+ lobar artery level. In addition, study quality was identified as a significant source of heterogeneity among studies on vessel-based analysis. And high-quality studies showed a much higher pooled sensitivity of 99% and specificity of 97%, which was considered adequate to confirm and rule out the presence of pulmonary embolism. Whereas patients with acute emboli are practically treated with anticoagulant therapy, regardless of burden of emboli, knowledge of the location and extent of disease in CTEPH helps in the selection of candidates for thromboendarterectomy[27]. Based on this purpose, CT can be used as a good tool for the diagnosis of CTEPH.

A higher but non-significant diagnostic performance was observed for promising CT in comparison with routine CT in overall arterials level. Dual-energy CT has two promising advantages. First, it may be used to provide a “one-stop” diagnostic assessment of CTEPH in anatomy and perfusion without any additional radiation exposure. Small occlusive clots sometimes have a greater influence on oxygenation changes than larger non-occlusive emboli. Lung perfusion imaging can increase the diagnostic performance of CTEPH by identifying those clots in the small vessels, which are difficult to visualize in CTPA. Functional information derived from perfusion imaging can offer a better representation of haemodynamic significance than CTPA alone[34]. Compared with routine CT, dual-energy CT (DECT) can provide an equivalent clinical information about lung perfusion at comparatively low dose. Second, kim et al reported that DECT can be used to differentiate between acute pulmonary thromboembolism and chronic pulmonary thromboembolism through the attenuation value of emboli, which is benefit to choose the correct treatment[35]. Additionally, 320-slices CT scanning can estimate pulmonary artery pressure (PAP) based on interventricular septum (IVS) curvature and evaluate pulmonary hemodynamics in patients with CTEPH[24]. Based on all the aspects mentioned above, DECT and 320-slices CT are promising tools in diagnosing CTEPH.

The sensitivity of the study by Tunariu et al. was much lower than that of the other studies [21]. The reason was thought to be that some patients with subsegmental pulmonary embolism were included in this study[13]. With the emergence of BPA, subsegmental arterials become increasingly important. BPA is implemented as an alternative interventional strategy for patients without surgical potential due to distal pulmonary artery obstructions with surgically inaccessible or comorbid illness[36]. Since studies included in our meta-analysis paid insufficient attention to subsegmental arterials, we can't extracted the data in subsegmental level.

Our meta-analysis has following potential limitations. First, the number of included studies was insufficient. This might reduce the statistical power of meta-analysis. Second, our meta-analysis combined results from trials with different CT techniques, which may lead to bias. Third, patients referred for suspected or confirmed CTEPH may lead to bias although subgroup analysis revealed no significant effect of patient selection. Fourth, the reference standards of included studies referred as DSA or V/Q scanning influence the reliability of the pooled data. Fifth, although subgroup analyses were conducted in overall arterials, some potential factors might be missed such as the contrast agent and the prevalence. Meta-regression wasn't conducted in patient-basis, main+ lobar arterials and segmental arterials because of insufficient trials. Sixth, diagnostic value of CTEPH in subsegmental arterials wasn't evaluated due to lack of data.

In conclusion, CT is a favorable method to rule in CTEPH and to rule out pulmonary endarterectomy (PEA) patients for proximal branches. Furthermore, dual-energy and 320-slices CT can increase the sensitivity for subsegmental arterials, which are promising imaging techniques for balloon pulmonary angioplasty (BPA) approach. In the near future, CT could position itself as the key for screening consideration and for surgical and interventional operability.

## Supporting Information

S1 Table PRISMA checklist. (PDF)
